# Main pancreatic duct involved IPMN without high-risk factors: how to judge the degree of malignancy based on MPD dilation?

**DOI:** 10.1097/MD.0000000000039323

**Published:** 2024-08-16

**Authors:** Yong Zhu, Yingfan Mao, Jianhua Wang, Zhongqiu Wang, Xiao Chen

**Affiliations:** a Department of Radiology, Jiangsu Province Hospital of Chinese Medicine, Affiliated Hospital of Nanjing University of Chinese Medicine, Nanjing, Jiangsu Province, China; b Department of Radiology, The Second Affiliated Hospital of Nanjing Medical University, Nanjing, Jiangsu Province, China.

**Keywords:** intraductal papillary mucinous neoplasm, magnetic resonance cholangiopancreatography, main pancreatic duct, malignant

## Abstract

The aim of this study was to evaluate the cutoff value for identifying malignance in main pancreatic duct (MPD)-involved intraductal papillary mucinous neoplasm (IPMN) with an MPD diameter ranging from 5 to 10 mm. Clinical-radiological characteristics of 142 patients, including MPD-involved IPMNs (n = 53) and branch-duct (BD)-IPMNs (n = 89) were analyzed. Logistic regression analysis was used to determine the risk factors of malignant IPMNs and invasive carcinoma. ROC curves were used to identify different cutoffs in terms of preoperative MPD values to predict the presence of invasive carcinoma as well as malignant IPMNs, and the prediction performance was evaluated. For MPD-involved IPMNs (5 mm < MPD < 10 mm), MPD diameter of 7.5 mm for discriminating malignant IPMNs (area under curve [AUC] = 0.67) and 7.7 mm for discriminating invasive IPMNs (AUC = 0.56) were found to be the optimal cutoff values at receiver operating characteristic curve (ROC) analysis. MPD > 7.5 mm and carbohydrate antigen19-9 (Ca19-9) > 37 U/ml were found to be predictors of malignant IPMNs at univariate, and MPD > 7.5 mm was a predictor in multivariate analysis in MPD-involved IPMNs. The AUC of the ROC curve of MPD (7.5 mm) combined with Ca19-9 in identifying malignant IPMNs was 0.73 in MPD-involved IPMNs. MPD (7.5 mm) combined with Ca19-9 performed well in identifying malignant IPMNs in MPD-involved IPMNs.

## 1. Introduction

Intraductal papillary mucinous neoplasm (IPMN) of the pancreas are tumors that grow in the main pancreatic duct (MPD) or branch duct (BD) with different papillary features and atypical mucin production, as well as segmentary or diffuse dilatation of the MPD, cystic dilatation of the secondary branch, or both.^[1]^ MPD-involved IPMNs were defined as a dilation of the MPD **≥** 5 mm, either segmental or diffuse, without identifiable reason for a duct obstruction and with or without signs of BD dilation. BD-IPMN was defined as a lesion characteristic for IPMN with a clear BD connection to the nondilated MPD. The diagnosis of IPMNs has increased dramatically in recent years.^[2–4]^ Accurate assessment of the risk of malignancy in such patients is a major clinical need so that surgical resection can be performed in the appropriate population. According to the 2010 World Health Organization classification, pancreatic IPMNs can be subcategorized into IPMNs with low- or moderate-grade dysplasia (benign) and IPMNs with high-grade dysplasia or those associated with invasive cancers (malignant).^[5,6]^ International guidelines have identified high-risk features (HRS) and worrisome features (WFs) as indications for pancreatectomy.^[5,7]^

WFs include 5 to 9 mm MPD, which allows for nonsurgical treatment of these patients, but this strategy has been challenged. Hackert et al^[8]^ reported that the malignant rate of IPMNs with MPD of 5 to 9 mm was 59%. In addition, Del et al^[9]^ found that in a large resection IPMN cohort MPD of 5 to 9 mm was the best predictor of high-grade dysplasia and invasive cancer.

Invasive cancer has also been reported in MPD patients with a small diameter and no nodules or symptoms.^[10–12]^ To date, there are few data on the malignant risk of IPMN with MPD diameters of 5 to 10 mm.^[13–15]^ How to judge the malignant degree of MPD-involved IPMN patients without high-risk factors? We hypothesize that even without high-risk factors, benign and malignant MPD-involved IPMNs still have significant differences in MPD diameter.

Thus, the aim of our study was to evaluate the diagnostic performance of MPD (5.0 mm) and carbohydrate antigen19-9 (Ca19-9) in identifying malignant IPMNs and invasive IPMNs in all types of IPMNs by using magnetic resonance imaging with magnetic resonance cholangiopancreatography (MRCP). In addition, for MPD-involved IPMNs with an MPD diameter ranging from 5 to 10 mm, we attempted to evaluate the cutoff value for further identifying malignant IPMNs.

## 2. Materials and methods

The local institutional review board approved this study, and patient informed consent was waived due to its retrospective nature.

### 2.1. Patients

This study was conducted in patients with pathologic examination–proven IPMNs from January 2017 to June 2022. The inclusion criteria were: with a final diagnostic result that was through surgery (and subsequent pathologic examination); with identification of low-, intermediate-, or high-grade dysplasia, or invasive carcinoma according to pathological results; and with MRCP examinations of the abdomen performed within the 14 days before treatment. The exclusion criteria were: with a history of benign and malignant tumors of ampulla or pancreas; with MPD diameter ≥ 10 mm in MPD-involved IPMNs; poor MRCP image quality due to artifacts or confusing location of the lesion. A total of 142 patients (58 men and 84 women), including MPD-involved IPMNs (n = 53) and BD-IPMNs (n = 89), were enrolled in the study.

### 2.2. Imaging techniques

All MRCPs were performed on a 1.5 T scanner (Phillips Intera Release 2.6, Phillips Healthcare, Best, The Netherlands) and a 3.0 T scanner (Ingenia 3.0T, Philips Healthcare, Best, The Netherlands) using a torso phased array coil. No oral or contrast agent was administered. Twelve thick slabs (40 mm sections) equally angulated at the coronal and sagittal planes were obtained during breath-hold. A fat-suppressed single-shot turbo spin/echo (TSE) sequence was used. Scan parameters were repetition time/echo time 8000/800 ms, field of view 300 mm, and in-plane resolution (frequency × phase) 0.94 mm × 1.17 mm. Maximum intensity projection images were reconstructed on the console and sent to the picture archiving and communication system. Single-shot TSE T2-weighted axial images of the liver were also included. Scan parameters were TE 80 ms, slice width/gap 7.0 mm/1 mm, and SENSE 2, with an in-plane resolution of 1.47 mm × 1.80 mm.

### 2.3. Imaging analysis and data collection

Two radiologists (with 7 years of experience in abdominal radiology, respectively), who were blinded to clinic-pathological information, interpreted MRCP images independently. If there was inconsistency, the consensus was achieved through discussion or referral to a third radiologist (with 20 years of experience in abdominal radiology).

Image features were analyzed on MRCP, including: MPD diameter (defined as the widest diameter of the MPD), location (head-neck/body-tail), and size (recorded as the maximum dimensions measured on cross-sectional images of preoperative T2-weighted images). Clinical symptom and biochemical data were collected from the medical records, including pancreatitis (with/without), Ca19-9, and carcinoembryonic antigen (CEA).

Each case was histologically classified according to the 2010 World Health Organization classification system for tumors of the pancreas.^[16]^ Low-grade dysplasia, intermediate-grade dysplasia, high-grade dysplasia, and invasive carcinoma were accurately described. For the purpose of our analysis, low- and intermediate-grade dysplasia were classified as benign, while high-grade dysplasia and invasive carcinoma were defined as malignant.^[17]^

### 2.4. Statistical analysis

Between MPD-involved IPMNs and BD-IPMNs, categorical variables were compared with using the χ^2^ test or Fisher exact test. Continuous variables were compared with using the Student *t* test or Mann–Whitney *U* test, when appropriate. WFs were retrospectively reclassified according to the 2017 updated version of the International Association of Pancreatology guidelines.^[7]^ In the present article, WFs included cyst > 3.0 cm, serum level of Ca19-9 > 37 U/mL, and MPD between 5 and 9 mm. Based on WFs, univariable and multivariable logistic regression analyses were used to determine risk factors of malignant IPMNs and invasive carcinoma. Then, receiver operating characteristic curve (ROC) curves were used to identify different cutoffs in terms of preoperative MPD values to predict the presence of invasive carcinoma as well as malignant IPMNs, and the prediction performance was evaluated by calculating the area under curve (AUC) value. All statistical analyses were carried out using SPSS software package version 22.0 (IBM Corp., Somers, NY, USA) and R software (R Foundation for Statistical Computing, version 3.4.1; https://www.r-project.org/). A two-sided *P* value less than 0.05 was considered to indicate statistical significance.

## 3. Results

### 3.1. Clinicopathological findings

A comparison of clinicopathological characteristics of patients with MPD-involved IPMNs (n = 53) and BD-IPMNs (n = 89) is shown in Table 1. Among MPD-involved IPMNs, 20 patients (20/53, 37.7%) were confirmed as malignant IPMNs and 14 patients (14/53, 26.4%) were confirmed as invasive carcinoma, with a significantly higher incidence than that in BD-IPMNs (both *P* < .001). However, there were no significant differences in tumor markers (including Ca19-9 and CEA) and other noteworthy clinical features (e.g., pancreatitis and location).

**Table 1:** Comparison of clinicopathological characteristics of patients with MPD-involved IPMNs and BD-IPMNs.

Characteristics	Total (n = 142)	MPD-involved IPMNs (n = 53)	BD-IPMNs (n = 89)	*P*
Age (range, years)	63.23 ± 9.20	64.91 ± 8.46	62.23 ± 9.51	.09
Gender (male/female)	58/84	22/31	36/53	.52
MPD diameter (range, cm)	0.46 ± 0.24	0.67 ± 0.18	0.32 ± 0.14	<.001
Location (head-neck/body-tail)	89/53	37/16	52/37	.12
Size (range, cm)	3.50 ± 2.14	3.71 ± 1.96	3.38 ± 2.45	.40
Ca19-9 (U/ml)	3.91 ± 1.14	35.33 ± 108.23	41.30 ± 118.43	.76
Ca 19-9 (>/≤ 37 U/ml)	23/119	9/44	14/75	.50
CEA (U/ml)	3.07 ± 1.94	3.30 ± 2.23	2.92 ± 1.74	.26
Pancreatitis (with/without)	4/138	0/53	4/85	.15
Lymph node invasion (with/without)	0/142	0/53	0/89	.14
Malignant IPMNs (with/without)	26/116	20/33	6/83	<.001
Invasive carcinoma (with/without)	17/128	14/42	3/86	<.001

Malignant IPMNs includes high intraepithelial neoplasia and invasive carcinoma.

Ca19-9 = carbohydrate antigen19-9, CEA = carcinoembryonic antigen, IPMN = intraductal papillary mucinous neoplasm, MPD = main pancreatic duct.

### 3.2. Risk factors for malignant IPMNs and invasive carcinoma

**Figure 1.**
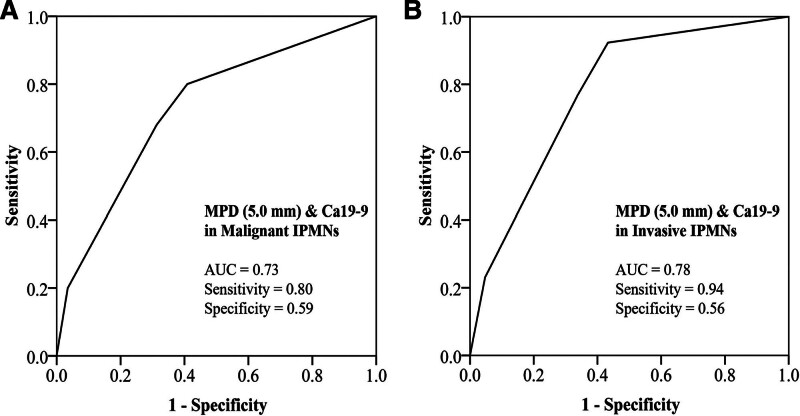
ROC curve of MPD (5.0 mm) and Ca19-9 in identifying malignant IPMNs (AUC = 0.73, sensitivity = 0.80, specificity = 0.59) (A) and invasive IPMNs (AUC = 0.78, sensitivity = 0.94, specificity = 0.56) (B). AUC = area under curve, Ca19-9 = carbohydrate antigen19-9, IPMN = intraductal papillary mucinous neoplasm, MPD = main pancreatic duct, ROC = receiver operating characteristic curve.

When 5 mm is selected as the cutoff value of MPD diameter, MPD > 5 mm (odds ratio [OR] = 7.71, *P* < .05) and Ca19-9 > 37 U/ml (OR = 3.13, *P* < .05) were found to be predictors of malignant IPMNs at univariate, and MPD > 5 mm was a predictor in multivariate analysis (OR = 5.81, *P* < .05) in all type IPMNs. Similarly, MPD > 5 mm was found to be a predictor of invasive carcinoma at univariate (OR = 7.00, *P* < .05) and multivariate analysis (OR = 8.16, *P* < .05) in this setting (Table 2). The AUC of the ROC curve of MPD and Ca19-9 in identifying malignant IPMNs and invasive IPMNs were 0.73 and 0.78, respectively (Fig. 1).

**Table 2:** The associated factors for malignant IPMNs and invasive IPMNs in all type IPMNs.

Characteristic	Malignant IPMNs				Invasive carcinoma			
	Univariate OR (95% CI)	*P* value	Multivariate OR (95% CI)	*P* value	Univariate OR (95% CI)	*P* value	Multivariate OR (95% CI)	*P* value
Age	1.0 (0.95–1.04)	0.786	0.98 (0.93–1.04)	0.779	1.03 (0.97–1.11)	0.395	1.04 (0.96–1.13)	0.883
Location (body-tail vs head-neck)	0.44 (0.17–1.18)	0.564	0.72 (0.24–2.20)	0.426	0.93 (0.29–2.93)	0.682	2.93 (0.68–12.67)	0.526
MPD (>5mm vs ≤ 5mm)	7.71 (2.85–20.86)	0.015	5.81 (1.95–17.33)	0.033	7.00 (1.86–26.41)	0.021	8.16 (1.70–39.27)	0.041
Ca19-9 (>37 U/ml vs ≤ 37 U/ml)	3.13 (1.15–8.53)	0.010	2.78 (0.73–10.52)	0.197	3.78 (1.11–12.87)	0.035	4.27 (0.77–23.80)	0.130
Size (>3.0 cm vs ≤ 3.0 cm)	1.78 (0.75–4.62)	0.256	1.85 (0.65–5.27)	0.620	0.62 (0.18–2.16)	0.285	0.37 (0.09–1.60)	0.216

Ca19-9 = carbohydrate antigen19-9, IPMN = intraductal papillary mucinous neoplasm, MPD = main pancreatic duct.

### 3.3. Risk factors in MPD-involved IPMNs

**Figure 2.**
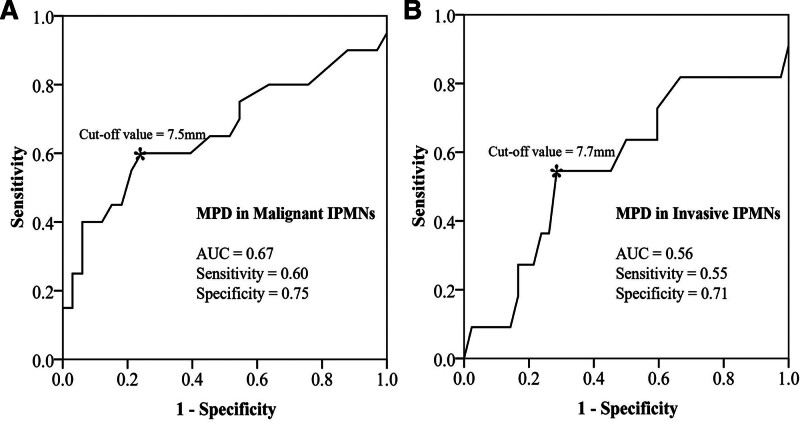
ROC curve to determine the cutoff values of MPD in identifying malignant IPMNs (AUC = 0.67, sensitivity = 0.60, specificity = 0.75) (A) and invasive IPMNs (AUC = 0.56, sensitivity = 0.55, specificity = 0.71) (B). The cutoff value was 7.5 mm for malignant IPMNs and 7.7 mm for invasive IPMNs. AUC = area under curve, IPMN = intraductal papillary mucinous neoplasm, MPD = main pancreatic duct, ROC = receiver operating characteristic curve.

For MPD-involved IPMNs (5 mm < MPD < 10 mm), an MPD diameter of 7.5 mm for discriminating malignant IPMNs (AUC = 0.67) and 7.7 mm for discriminating invasive IPMNs (AUC = 0.56) were found to be the optimal cutoff values at ROC analysis (Fig. 2).

**Figure 3.**
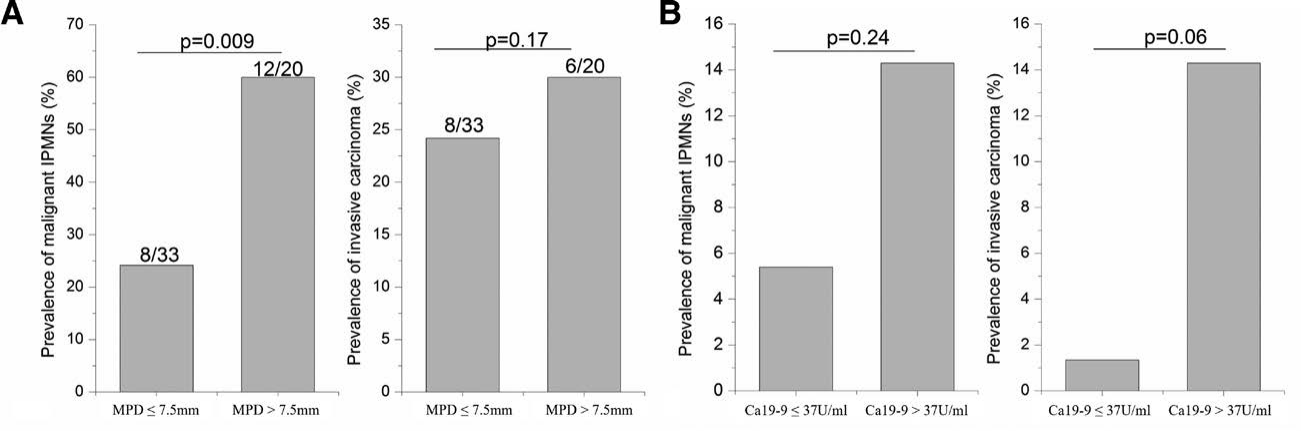
Prevalence of malignant IPMNs and invasive carcinoma in MPD-involved IPMNs based on MPD (7.5 mm) (A) and BD-IPMNs based on Ca19-9 levels (B). BD = branch duct, Ca19-9 = carbohydrate antigen19-9, IPMN = intraductal papillary mucinous neoplasm, MPD = main pancreatic duct.

When 7.5 mm is selected as the cutoff value of MPD diameter, 12 (12/20, 60.0%) MPD-involved IPMNs were confirmed as malignant IPMNs in MPD > 7.5 mm group, which was significantly higher than that in MPD ≤ 7.5 mm group (8/33, 24.2%) (*P* = .009) (Fig. 3).

MPD > 7.5 mm (OR = 4.69, *P* < .05) and Ca19-9 > 37 U/ml (OR = 4.92, *P* < .05) were found to be predictors of malignant IPMNs at univariate, and MPD > 7.5 mm was a predictor in multivariate analysis (OR = 7.36, *P* < .05) in MPD-involved IPMNs (Table 3).

**Table 3:** The associated factors for malignancy in MPD-involved IPMNs

Characteristic	Malignant IPMNs				Invasive carcinoma			
	Univariate OR (95% CI)	*P* value	Multivariate OR (95% CI)	*P* value	Univariate OR (95% CI)	*P* value	Multivariate OR (95% CI)	*P* value
Age	1.0 (0.93–1.06)	0.378	1.01 (0.92–1.11)	0.592	1.03 (0.97–1.11)	0.981	1.06 (0.94–1.19)	0.972
Location (body-tail vs head-neck)	0.26 (0.07–1.08)	0.838	0.36 (0.07–1.94)	0.284	0.93 (0.29–2.93)	0.258	1.99 (0.31–12.98)	0.864
MPD (>7.5 mm vs ≤ 7.5 mm)	4.69 (1.42–15.53)	0.011	7.36 (1.79–30.22)	0.036	2.40 (0.62–9.25)	0.495	4.07 (0.76–21.95)	0.125
Ca19-9 (>37 U/ml vs ≤ 37 U/ml)	4.92 (1.07–22.70)	0.042	2.10 (0.25–17.79)	0.190	3.78 (1.11–12.87)	0.049	1.41 (0.09–22.43)	0.682
Size (>3.0 cm vs ≤ 3.0 cm)	0.79 (0.24–2.54)	0.159	0.83 (0.20–3.47)	0.281	0.62 (0.18–2.16)	0.367	0.22 (0.04–1.30)	0.618

Ca19-9 = carbohydrate antigen19-9, IPMN = intraductal papillary mucinous neoplasm, MPD = main pancreatic duct.

**Figure 4.**
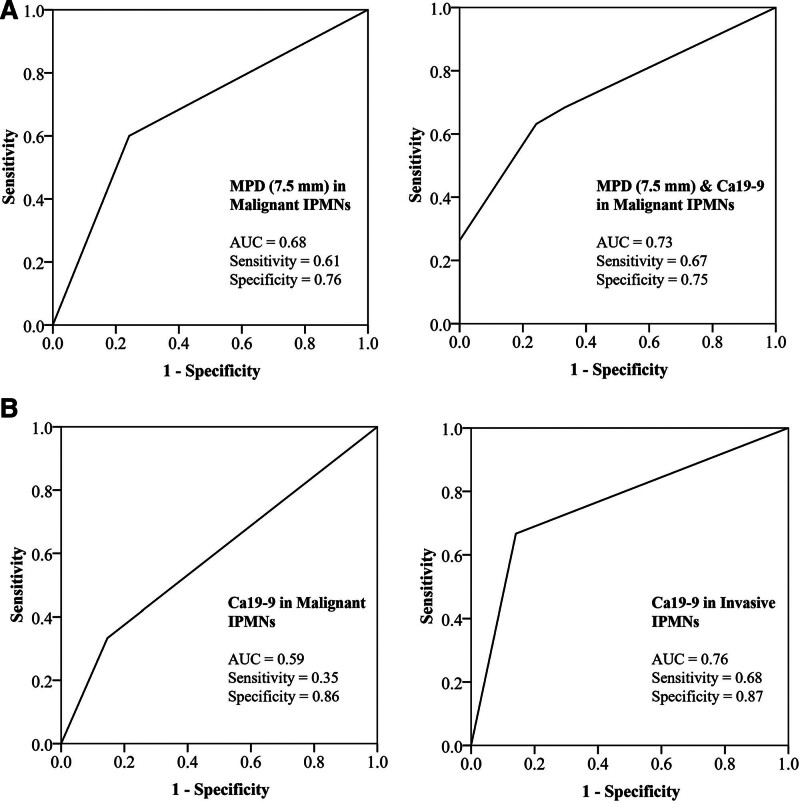
(A) ROC curve of MPD (7.5 mm) alone (AUC = 0.68, sensitivity = 0.61, specificity = 0.76) or combined with Ca19-9 (AUC = 0.73, sensitivity = 0.67, specificity = 0.75) in identifying malignant IPMNs in MPD-involved IPMNs. (B) Ca19-9 in identifying malignant IPMNs (AUC = 0.59, sensitivity = 0.35, specificity = 0.86) and invasive IPMNs (AUC = 0.76, sensitivity = 0.68, specificity = 0.87) in BD-IPMNs. AUC = area under curve, BD = branch duct, Ca19-9 = carbohydrate antigen19-9, IPMN = intraductal papillary mucinous neoplasm, MPD = main pancreatic duct, ROC = receiver operating characteristic curve.

The AUC of the ROC curve of MPD (7.5 mm) alone or combined with Ca19-9 in identifying malignant IPMNs were 0.68 and 0.73, respectively in MPD-involved IPMNs. The AUC of Ca19-9 in identifying malignant IPMNs and invasive IPMNs in BD-IPMN is 0.59 and 0.76, respectively (Fig. 4).

## 4. Discussion

MPD dilation is of great significance in judging the malignant degree of MPD in IPMN patients without high-risk factors. When 7.5 mm is selected as the cutoff value of MPD diameter, MPD combined with Ca19-9 performed well in identifying malignant IPMNs in MPD-involved IPMNs.

In our study, MPD of 5.0 mm and Ca19-9 level have certain values in identifying Ca19-9 malignant IPMNs and invasive IPMNs in all types of IPMNs. Even in the IPMN with an MPD size of 5 to 9 mm, the malignant risk has reached 59%. To date, few studies have been reported about the clinicopathological characteristics of MD and mixed-type IPMNs with an MPD size of 5 to 9 mm.^[8]^ The Sendai guidelines^[7]^ proposed categories of “worrisome features,” including imaging findings such as MPD dilation 5 to 9 mm, to improve specificity, but it has been proven to be unsatisfactory in clinical application.^[18]^ We believe that further subclassification of MPD diameters between 5 and 9 mm would provide additional benefits for risk stratification.

We found that for MPD-involved IPMNs (5 mm < MPD < 10 mm), an MPD diameter of 7.5 mm for discriminating malignant IPMNs (AUC = 0.67) was found to be the optimal cutoff value. Almost 12 (60.0%) MPD-involved IPMNs were confirmed as malignant IPMNs in the MPD > 7.5 mm group, which was significantly higher than that in the MPD ≤ 7.5 mm group (*P* = .009). Abdeljawad et al^[19]^ reported that when the diameter of the MPD is 5 to 9 mm, the cutoff value of 8 mm is helpful to distinguish benign and malignant MD-IPMNs, which is consistent with our conclusion to a certain extent. Crippa et al^[20]^ found that the risk of MPD ≤ 8 mm in the head and MPD ≤ 6 mm in the body/tail of IPMNs was lower in the absence of other WFs or high-risk stigmata. The association between MPD dilation and the risk of neoplasia has long been noted in retrospective cohort studies. Our study provides further evidence of what diameter MPD should be surgically treated.

In addition, MPD (7.5 mm) combined with Ca19-9 performed well in identifying malignant IPMNs in MPD-involved IPMNs. The current International Association of Pancreatology guidelines include elevated serum CA 19-9 as a “worrisome feature.” Ciprani found that elevated Ca19-9 levels were associated with malignancy in IPMN, as 63% of patients with Ca19-9 > 37 U/ml had either high-grade dysplasia or invasive carcinoma.^[21]^ Ca19-9 has been proven to be a predictor of malignancy in the presence of IPMN, especially closely related to invasive cancer.^[21,22]^

This study has some limitations. First, this is a single-center retrospective study. Further analysis based on a larger sample size and external validation is required to confirm our findings. Second, for the assessment of MPD-IPMN, the change of the MPD diameter during the disease progression may be valuable, which is not involved in this study due to its retrospective nature, and needs further verification. Nevertheless, our research has laid a foundation for future research on the subclassification of MPD diameters.

In conclusion, this study shows that further subclassification of MPD diameters ranging from 5 to 10 mm provides additional benefits for risk stratification. MPD (7.5 mm) combined with Ca19-9 performed well in identifying malignant IPMNs in MPD-involved IPMNs.

## Acknowledgements

The authors thank the Multidiscipline Team in Hepatobiliary Disease of the Second Affiliated Hospital of Nanjing Medical University for professional discussion for this manuscript.

## Author contributions

**Conceptualization:** Yong Zhu, Yingfan Mao, Xiao Chen.

**Data curation:** Yong Zhu, Jianhua Wang.

**Formal analysis:** Yong Zhu.

**Methodology:** Yong Zhu, Yingfan Mao, Jianhua Wang, Zhongqiu Wang, Xiao Chen.

**Writing—original draft:** Yong Zhu.

**Validation:** Yingfan Mao.

**Writing—review & editing:** Zhongqiu Wang, Xiao Chen.
